# The Poor-Law Infirmary

**Published:** 1907-06-22

**Authors:** 


### Poor-Law Department.

### THE POOR-LAW INFIRMARY.

### THE ADMISSION OF PATIENTS.

One of the unsatisfactory characteristics of Poor-law infirmaries as at present constituted is the method in which patients are admitted. Accidents are by right brought to the infirmary, and admitted at once, as they should be. These cases are often brought by the police, as are cases of wanderers found homeless and suffering, and also cases of acute alcoholism. And here may be noted the advantage possessed by the man who drinks himself into a state of madness which makes him dangerous to himself and others, over the man who merely gets drunk and incapable, or staggers about the streets in such a way as to attract police attention. The latter is taken up before a magistrate, charged and punished; while the "madly drunk," who is probably the more abandoned of the two, and undeniably the more dangerous, is carefully nursed back to health and then let go free, without punishment, if not without reproof. It would be only fair if he also was punished for his drunkenness after he had sufficiently recovered from the effects of it to realise his fault. This, however, is by the way.

With the admission of such exceptional cases to the infirmary there is no fault to find. But it is different when we come to ordinary medical cases, and, above all, when we have to deal with chronic illnesses. Ordinary cases as a rule obtain an infirmary order on the recommendation of the parish medical officer, a gentleman whose fitness to do so cannot be disputed. But unfortunately there is sometimes room for the complaint that the practitioner is too ready to send to the infirmary cases which, if not admitted, would demand a prolonged attendance from him. Occasionally guardians complain that if the medical officer were paid for each visit to his pauper patients he would not be so ready to suggest their transference to the wards of the infirmary. Certainly the practitioner might reasonably plead that in that institution the patient will receive better nursing and possibly better food than in his own home. Assuredly he will have more air and will be free from the assiduities of well-meaning but unwise friends. This is a perfectly comprehensible reason for sending a patient to the infirmary, but it cannot be accepted as valid unless the disease is so acute that there is a reasonable probability that he will not recover elsewhere. The test question which a conscientious medical officer should put to himself is whether or not he would advise removal to a hospital of a similar case among his poor non-pauper patients. Indeed, in the case of the pauper the moral responsibility resting upon him is greater, because of the weight attaching to his recommendation, which makes it a very difficult thing for the medical superintendent of the infirmary to refuse the case if by any means he can find room for it. The medical superintendent who declined to take in a case because, in his opinion, it did not need infirmary treatment would be certain to hear of it at the next board meeting, for it would not be very rash to say that every pauper now-a-days has a guardian willing to champion his demands against the officials of the union, and that every medical officer can find one to take up the cudgels for him against the superintendent of the infirmary.

In the matter of infirmary admission guardians are, in fact, too obliging. They make a merit of being kind to the poor. "I thought it likely that the poor fellow would be better looked after in the infirmary than at home," is a frequent explanation why a guardian presses the admission of a case which, on medical grounds, might very well be refused. And the amiable gentleman does not perceive that by voting for the admission of such a case, he is lessening the accommodation available for others whose home conditions may be no better, while their state of health may be such as to make infirmary treatment an imperative necessity. Of course the medical superintendent may protest that the wards are over-crowded, and if the Local Government Board inspector happens to visit them at the time, a sharp letter complaining of the condition of affairs may be sent to the clerk to the guardians. In a voluntary hospital such a state of things would at once result in instructions being given that care should be taken to admit only acute cases, though an appeal might be made to the public to give money for an extension of the building. But in the rate-supported institution the affair is much simpler. A letter to the Local Government Board to explain that the demand upon the accommodation of the infirmary is greater than its resources—a statement which it is easy to substantiate by the statistics of applications and the number of beds; permission asked to add to the accommodation, and, in view of the statistics, granted; and at once additions may be begun without even the formality of an appeal to the rate-payers, who have to bear the cost.

It might be thought that the fact that the guardians have the right to make a charge for the accommodation and treatment given at the infirmary would act as a check upon abuse of this sort. But it does not. The genuinely poor are admitted, of necessity, free. When payment is demanded it is often less than the cost of maintenance, a fact which the patient's friends fully appreciate. They get rid of the trouble of nursing the case and save money into the bargain. Occasionally the full cost of maintenance may be charged, and when this is done the finances of the family are sure to be such that it is not right that a member of it should seek the accommodation provided for paupers. In an occasional case the admission may be justified, and in a chronic one where the patient has not relatives to nurse him it may be excused, but there is a tendency among a class of people who have a sufficient income but insufficient self-respect, to slide away from the responsibility of their sick and aged kinsfolk, and leave them stranded, poor human derelicts, in the wards of the workhouse infirmary. A considerable number of the infirm must of necessity end their days there, because they have none to care for them, but that makes it all the more desirable that the room which these may need should not be taken up by persons whose families should look after them. Moreover, the larger infirmaries now aspire to be training schools for nurses, and, indeed, they provide an admirable ground for such work; but it is, therefore, all the more important that a great part of the space available should not be given up to chronic cases which require kindly attention rather than skilled nursing. For such the infirm wards of the workhouse are the proper place. For as the Poor-law infirmaries have more and more taken up the work and the position of general hospitals, the objection to them among possible patients who differentiate sharply between "the house" and "the infirmary" has greatly decreased.

We have spoken of the paying patients in the infirmaries, and have said that when people can afford to pay for their maintenance there, only the strongest medical reasons can justify their presence. But it must be remembered that when a patient gets into the infirmary on a paying basis, it is very difficult to get him out, even though the payments cease. In fact, one might say that his claim to the accommodation of the infirmary is all the stronger if he is destitute. Once a chronic patient is admitted, on whatever basis, he is there for life. And it is peculiar, and worthy of note, that a patient may be admitted on the promise of payment—demanded because the relieving officer reports that the family income is a good one—and for the guardians to find that his children all lose their employment within a month or two! The payments fall into arrear, and when an explanation is demanded a terrible tale of poverty is told, the arrears are marked off, and the future payment is fixed at a lower sum, or excused altogether. And, of course, the patient remains where he is, and the chances are that, however much the family fortunes may improve—as they often do with suspicious promptness—the contribution will never be renewed. In short, Poor-law infirmaries are abused as much as voluntary hospitals, and as they are maintained by compulsory contributions from all the self-supporting classes of the community, this abuse constitutes a grievous wrong.

